# Cost-analysis of total knee arthroplasty with robot-assisted surgery versus conventional approach

**DOI:** 10.1007/s11701-026-03508-0

**Published:** 2026-05-30

**Authors:** Bogdan Sorin Capitanu, Serban Dragosloveanu, Eduard Gabriel Botnariu, Mihnea-Valentin Ionescu, Elena Druica, Dana-Georgiana Nedelea, Cristian Scheau

**Affiliations:** 1 The “Carol Davila” University of Medicine and Pharmacy, Bucharest, 050474 Romania; 2 Traumatology and Osteoarticular TB, “Foisor” Clinical Hospital of Orthopaedics, Bucharest, 021382 Romania; 3 Department of Applied Economics and Quantitative Analysis, University of Bucharest, Bucharest, 030018 Romania; 4 Titu Maiorescu University, Bucharest, 040056 Romania

**Keywords:** Robotic-assisted surgery, Cost analysis, Healthcare economics, Total knee arthroplasty, Economic burden

## Abstract

Total knee arthroplasty (TKA) volume is rising, and robotic assistance is promoted for greater precision and faster recovery; however, cost data from Romania remain limited. This retrospective cohort study analysis examined 400 consecutive primary TKAs: 100 robotic-assisted (rTKA) and 300 manual (mTKA). Outcomes included operating room (OR) time, length of stay (LOS), 90-day events, and total in-hospital costs broken down by category. Cost determinants were explored using LASSO with cross-validation and corroborated by ordinary least squares models. rTKA was associated with longer OR time (103.55 ± 23.92 vs. 80.29 ± 17.53 min) and longer LOS (5.99 ± 1.46 vs. 4.79 ± 1.35 days). The subvastus approach correlated with shorter LOS. Hospitalization costs reflected the interplay of perioperative care and institutional practice rather than the surgical approach alone: higher expenditures tracked most strongly with ward medications and sanitary materials, robotic use itself, longer OR time, and ICU consumables, whereas the subvastus approach tended to lower costs. Model fit was moderate. Within 90 days, rTKA had one non-injurious fall unrelated to implant stability; mTKA had two wound dehiscences (one readmission) and one transient stiffness that resolved with rehabilitation. In this setting, rTKA was associated with higher hospitalization costs despite low early complication rates in both groups. Improving specific steps before, during, and after surgery could reduce the extra costs of robotic TKA without harming results. To know the true value, larger multi-hospital studies with longer follow-up, detailed cost breakdowns, and patient-reported outcomes are needed.

## Introduction

Total knee arthroplasty (TKA) is an effective treatment for patients with advanced symptomatic knee osteoarthritis, aiming to restore knee mobility, relieve pain, and enhance the quality of life [1, 2]. Due to rising obesity rates and increased life expectancy, the demand for TKA is expected to grow significantly, by an estimated 139% by 2040 and 469% by 2060 [3]. Although TKA has consistently demonstrated favorable functional and radiological outcomes over time, approximately 20% of patients remain dissatisfied with the results, showing the need for continued improvement in surgical techniques and patient-specific approaches [4–6].

Additionally, a study by Heo et al., which analyzed complications within six months following conventional TKA, found that 14.4% of patients experienced major complications, while 46.6% developed minor complications such as joint stiffness and swelling. Major complications were defined as those requiring reintervention or reoperation [7]. These adverse events not only contribute to increased patient morbidity and mortality, but also significantly raise hospitalization costs, representing a substantial burden on the healthcare system.

Robotic-assisted total knee arthroplasty (rTKA) aims to improve surgical precision and minimize complications that can elevate healthcare costs [8]. Research by Mulpur et al. showed that robotic-assisted procedures are linked to improved patient satisfaction and quicker achievement of independent ambulation compared to manual techniques [9]. Stauss et al. found that rTKA decreases surgical time, shortens length of hospital stay (LOS), and reduces 90-day complication and readmission rates [10]. Similarly, Constantinescu et al. found that rTKA leads to a reduction in perioperative complications and promotes faster recovery [11]. These findings highlight the advantages of robotic-assisted techniques in rTKA, particularly their ability to improve patient outcomes by reducing complication rates. rTKA offers consistent and predictable component alignment along with optimized soft tissue balancing, which not only enhances clinical outcomes but also contributes to a lower incidence of postoperative complications. Furthermore, this reduction in complications may help lower the financial burden of postoperative care, supporting a more efficient allocation of healthcare resources [12].

To our knowledge, the existing literature lacks data on the cost analysis of rTKA in Romania. A review of the international literature identified several studies from the United States. One such article by Hua et al. used a decision-analytic model to evaluate the economic impact of rTKA. Their findings suggest that, in high-volume hospitals, despite the substantial initial investment in technology and personnel training, these costs may be offset by lower complication and revision rates [13]. Similarly, a study by Ong et al. reported that rTKA was associated with reduced length of stay, lower index hospitalization costs, and fewer complications within 90 days postoperatively, supporting its potential economic advantage [14]. Despite these important findings, when it comes to what factors contribute most to the number of hospitalization days, or hospitalization costs, the literature is scarce.

Given the increasing adoption of this technology and the current lack of national data, the present study aims to compare rTKA with manual total knee arthroplasty (mTKA) in terms of operating room time, hospital length of stay, and total hospitalization costs. Secondary objectives include evaluating the 90-day postoperative complication rate and readmission rate.

## Materials and methods

### Study design

This retrospective cohort study was conducted at the “Foisor” Clinical Hospital of Orthopaedics, Traumatology, and Osteoarticular Tuberculosis. Ethical approval was obtained from the hospital’s Ethics Council (no. 11326/10.10.2024). Written informed consent was provided by all participants on admission, and the study complied with the ethical principles of the Declaration of Helsinki (1964) and its subsequent amendments.

### Study group

Between May 2023 and October 2024, a total of 400 patients who met the inclusion criteria were included in the study. Participants were divided into two groups: rTKA group with 100 patients who underwent robotic-assisted total knee arthroplasty and mTKA with 300 patients who underwent manual total knee arthroplasty. To ensure comparability between the two groups, propensity score matching was employed.

The rTKA cohort was first established by consecutively including all eligible patients treated during the study period. Subsequently, propensity score matching was performed using a larger institutional retrospective database of approximately 1,200 patients who underwent mTKA. From this broader cohort, matched controls were selected using a 1:3 nearest-neighbour matching algorithm based on predefined demographic and clinical covariates, including age, sex, body mass index, diabetes mellitus status, preoperative alignment parameters (aHKA and JLO), and surgical approach.

The purpose of matching was to generate comparable study groups and minimize selection bias. All primary comparative analyses were performed on the matched cohorts. Post-matching baseline characteristics confirmed satisfactory balance between groups.

### Inclusion and exclusion criteria

The study included patients with symptomatic advanced knee osteoarthritis, classified as Kellgren–Lawrence grade 3 or 4 on radiological examination, who were scheduled for and underwent primary TKA in our clinic. Exclusion criteria were: patients with complications unrelated to surgery, patients with congenital lower limb deformities requiring special implants, and patients who did not have a minimum follow-up of 90 days postoperatively.

### Surgical protocol

All procedures were performed in a high-volume arthroplasty center by experienced knee replacement surgeons, consistent with the specialized profile of our institution. Each participating surgeon had a minimum of 5 years of experience in knee arthroplasty. Overall, a total of 13 surgical teams contributed cases to the study. Within the robotic-assisted TKA group, cases were distributed among these teams, with the number of robotic procedures per team ranging from 2 to 15 during the study period. This multi-surgeon design reflects real-world clinical practice while maintaining a consistent institutional standard of care and perioperative management.

All patients, in both the manual and robotic-assisted groups, underwent the same standardized preoperative radiographic protocol consisting of bilateral standing full-length lower-limb radiographs and lateral knee radiographs. Radiographic magnification was standardized using a calibrated 25-mm reference ball. Preoperative planning was mandatory for every case and was performed using MediCAD (version 7.0). Although the ROSA system used in our clinic operates in an imageless configuration and therefore does not require preloaded imaging data, preoperative planning was routinely conducted to optimize surgical preparation, confirm implant sizing and alignment strategy, ensure implant and instrument availability, and provide an additional verification step before surgery. As both study groups followed the identical imaging and planning protocol, no additional imaging-related costs were incurred for the robotic-assisted cohort.

For patients who underwent mTKA, a cemented posterior-stabilized prosthesis was used (NexGen^®^ LPS; Zimmer Biomet, Warsaw, IN, USA). Mechanical alignment was performed using either a subvastus or a medial parapatellar approach, depending on the surgical team’s preference.

In the rTKA group, a subvastus or medial parapatellar approach was also used. However, a personalized kinematic alignment technique was applied. Cemented implants (Zimmer NexGen or Zimmer Persona) were selected based on the availability of the robotic kit. The robotic system used was the ROSA^®^ Knee robot, developed by Zimmer Biomet in collaboration with MedTech. This imageless interactive robotic platform uses a robotic arm to position the cutting guides accurately according to an intraoperative navigation plan. It is important to note that the surgeon remains responsible for the surgical approach, placement of retractors, and bone cuts, as the system assists but does not automate the surgical procedure. The technique used followed the manufacturer’s recommended ROSA^®^ surgical protocol.

All patients received preoperative antibiotic prophylaxis consisting of a single 1 g dose of vancomycin and two doses of cefuroxime at 1.5 g each. To prevent deep vein thrombosis (DVT), patients received prophylactic treatment with low-molecular-weight heparin (LMWH), specifically Clexane (4000 IU). Administration began 12 h after spinal anesthesia and was repeated every 24 h. This regimen continued until discharge and was maintained for 28 days post-discharge. After the resolution of anesthesia, all patients began active physical therapy and continued with home-based exercise for six weeks, under follow-up supervision to ensure adherence.

### Outcome measures

Patients were followed for a period of 90 days to monitor complications related to surgery, such as symptomatic DVT or pulmonary embolism, wound complications (including dehiscence, infection, or necrosis), and reinterventions due to septic events or implant malposition. Additional outcomes assessed included hospital length of stay, in-hospital mortality, and total hospitalization costs for all patients included in the study.

Hospital costs were analyzed by categorizing them based on timing, preoperative or postoperative (within 90 days, including readmissions) and by medical department, including the Intensive Care Unit (ICU), operating room (OR), and current admission ward. Each of these sections was further broken down into costs related to medication, paraclinical investigations, and sanitary materials. Additional costs included laboratory investigations, inpatient care, and meals during hospitalization. The choice of implant depended on the surgical team or the availability of a specific system or kit at the time of surgery.

### Data analysis

The analysis was conducted from a hospital perspective and focused on direct in-hospital costs recorded at the patient level. All statistical analyses were performed using R software (version 4.3.1.). Continuous variables were summarized as means ± standard deviations (SD), and categorical variables as counts and percentages. Baseline characteristics (age, BMI, alignment parameters) and perioperative outcomes (operating room time, hospital stay) were compared between rTKA and mTKA groups using independent samples t-tests for continuous variables and chi-square (χ²) tests for categorical variables. A two-sided *p* < 0.05 was considered statistically significant.

This study employed the least absolute shrinkage and selection operator (LASSO) regression with four-fold cross-validation as a variable selection and regularization technique to identify a parsimonious set of predictors associated with hospitalization costs. LASSO is widely used in medical statistics for variable selection, as it reduces model complexity and limits overfitting by shrinking less informative coefficients towards zero [15]. Compared with traditional stepwise regression, LASSO provides improved stability and is less sensitive to random variation in the data [16]. Based on this methodology, we aimed to screen and select relevant predictors of hospitalization costs. Variables retained through the LASSO procedure were subsequently examined using ordinary least squares (OLS) multiple regression models, to estimate the direction, magnitude and statistical significance of associations. This two-step approach allowed us to combine data-driven variable selection with interpretable effect estimation.

To further examine how predictors affected specific categories of hospitalization costs, we estimated separate multiple linear regression models for each cost type. Continuous predictors were standardized prior to model estimation to allow comparability of effect sizes, while categorical variables were entered in their original form. Also, prior to modeling, the distribution of each cost component was visually and statistically assessed. For outcomes that exhibited non-normal distribution, a natural logarithmic transformation was applied to improve linearity and homoscedasticity of residuals. These included Intensive Care Unit (ICU) medication (ICU_med), ICU sanitary materials (ICU_sanitary_mat), Operating Room (OR) medication (OR_med), OR sanitary materials (OR_sanitary_mat), OR sanitary materials without prosthesis (OR_sanitary_mat_no_impl), and Ward medication (Ward_med) and sanitary materials (Ward_sanitary_mat). All the available predictor variables were entered simultaneously into each model, and interaction terms (e.g., BMI × Robotic surgery) were included where theoretically justified. The final models were selected using stepwise regression. Model diagnostics were used to assess multicollinearity, residual normality, and heteroskedasticity. Model fit for OLS regression was evaluated using R² and adjusted R² values, while cross-validated out-of-sample R² for LASSO regression was used as an additional measure of predictive robustness. Statistical significance was defined as *p* < 0.05.

The distinct variable selection strategies were tailored to respond to different objectives of each model. LASSO regression was applied to the total cost model to obtain a parsimonious and stable set of predictors and reduce the risk of overfitting. In contrast, for component-specific models, stepwise regression was used to allow a more flexible exploration of the heterogeneous and potentially weaker associations underlying individual cost categories. Given the relatively low explanatory power of component-specific models, a more flexible selection approach was preferred to retain potentially weak but informative associations.

## Results

A total of 400 patients were included, with 300 undergoing mTKA and 100 rTKA. Baseline characteristics were similar between groups regarding age, sex, BMI, diabetes, and alignment parameters (Table 1).

**Table 1:** Summary of Patient Characteristics, Surgical Approach, and Clinical Data

Parameter	rTKA	mTKA	*P*-value
Age (years)	69.02 ± 5.25	68.99 ± 6.61	0.9632
Gender (M/F)	27/71	71/229	0.5913
BMI (kg/m^2)^	31.01 ± 4.75	30.67 ± 4.05	0.5270
Diabetes mellitus (Y/N)	26/69	74/231	0.6349
aHKA (degrees)	-2.40 ± 4.94	-3.28 ± 4.94	0.1238
JLO (degrees)	175.73 ± 3.98	174.86 ± 4.09	0.0602
Surgical approach	MPA: 46 SVA: 54	MPA: 143 SVA: 157	0.8623
OR time (mins)	103.55 ± 23.92	80.29 ± 17.53	*< 0.0001*
Hospital stay (days)	5.99 ± 1.46	4.79 ± 1.35	*< 0.0001*
Hospital stay (days)	MPA: 6.52 ± 1.50 SVA: 5.53 ± 1.27	MPA: 5.13 ± 1.29 SVA: 4.47 ± 1.33	*< 0.0001* *< 0.0001*

Statistically significant results are shown in italics

MPA – medial parapatellar approach; SVA – subvastus approach

**Fig. 1:**
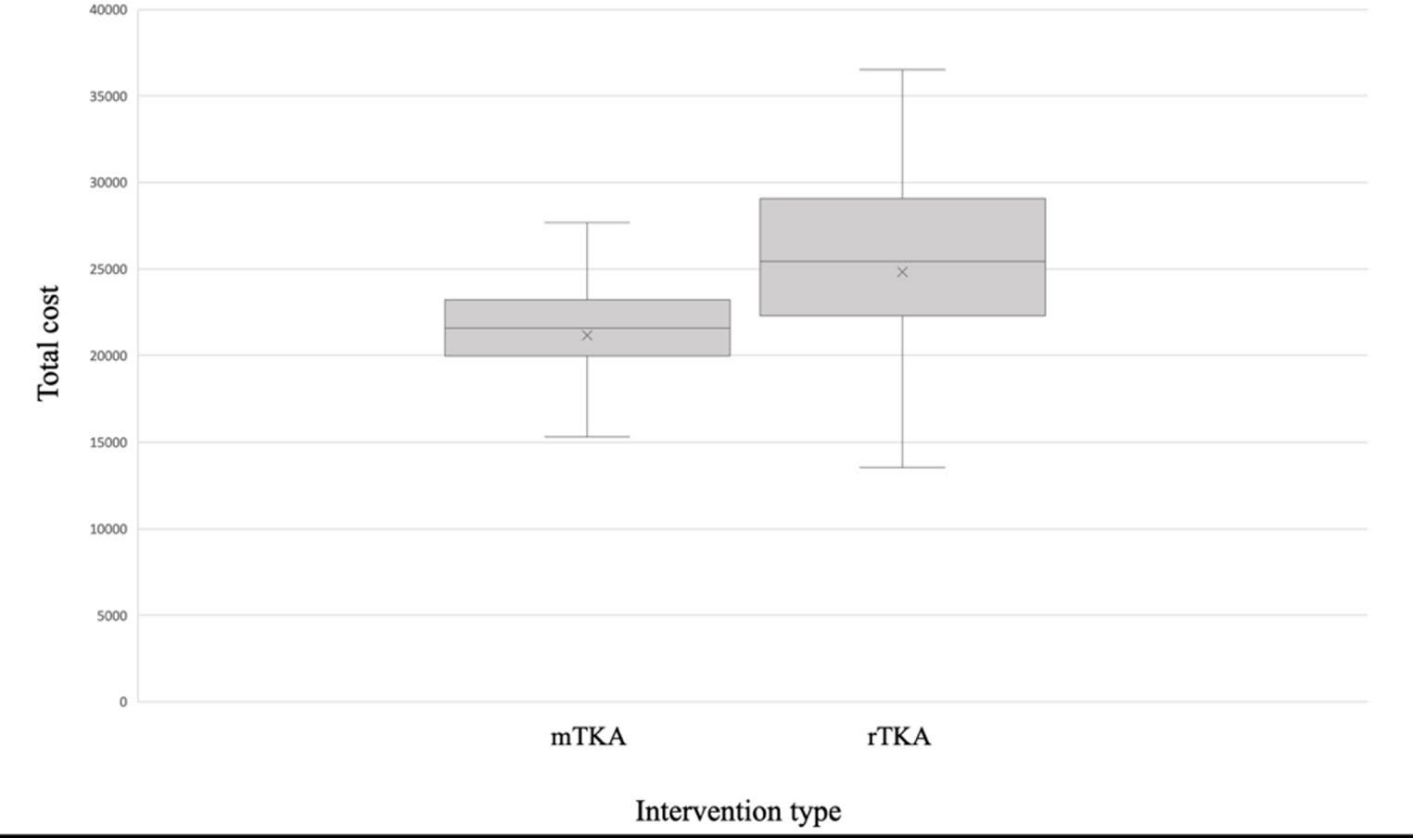
Boxplot of total hospitalization costs stratified by rTKA vs. mTKA

OR time and length of hospital stay were significantly longer in rTKA compared to mTKA. When analyzed by surgical approach, the subvastus approach was associated with a shorter length of hospital stay. In the mTKA group, the mean cost was 21176.30 ± 3,767.81 RON (median 21575.33 RON), whereas in the rTKA group the mean was 24821.50 ± 6449.32 RON (median 25451.81 RON) (Fig. 1). Regarding complications, within the 90-day follow-up the rTKA group had a single event, a same-level fall due to tripping, with no injury and unrelated to the stability of the operated knee. In contrast, the mTKA group recorded three events: two cases of wound dehiscence and one case of limited range of motion (ROM). The patient with limited ROM ultimately regained satisfactory motion after an intensive rehabilitation program; implants were optimally positioned with full postoperative extension, and the initial stiffness was likely related to nonadherence to the rehabilitation protocol.

### Predictors of total hospitalization costs

**Fig. 2:**
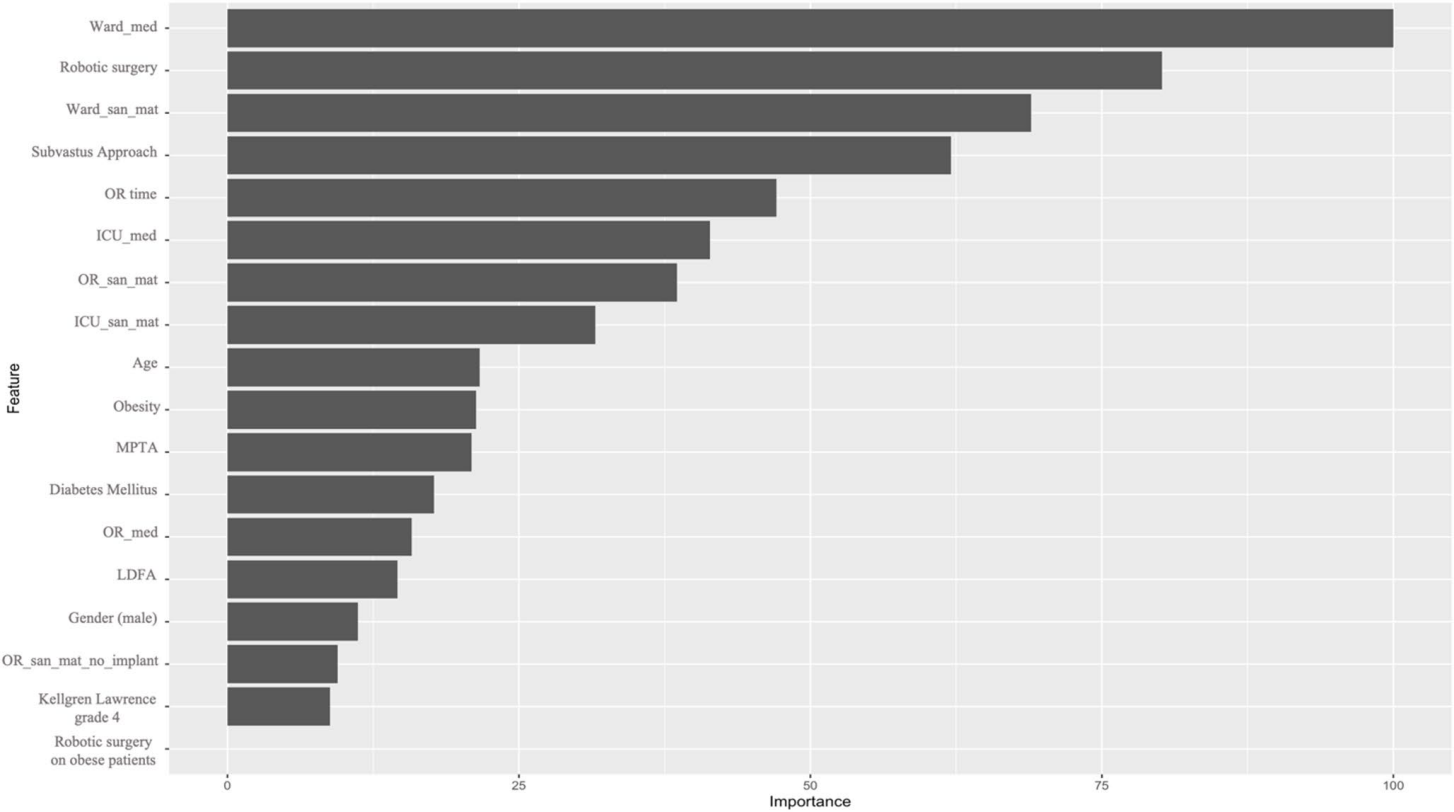
Variables retained by LASSO regression

Regression analyses were conducted in a two-step approach. First, LASSO regression with cross-validation was used to identify a parsimonious set of predictors associated with hospitalization costs (Fig. 2). Second, these predictors were examined using ordinary least squares (OLS) regression models to estimate the direction, magnitude, and statistical significance of associations. Standardized coefficients are reported for continuous variables, while coefficients for categorical predictors remain unstandardized.

**Fig. 3:**
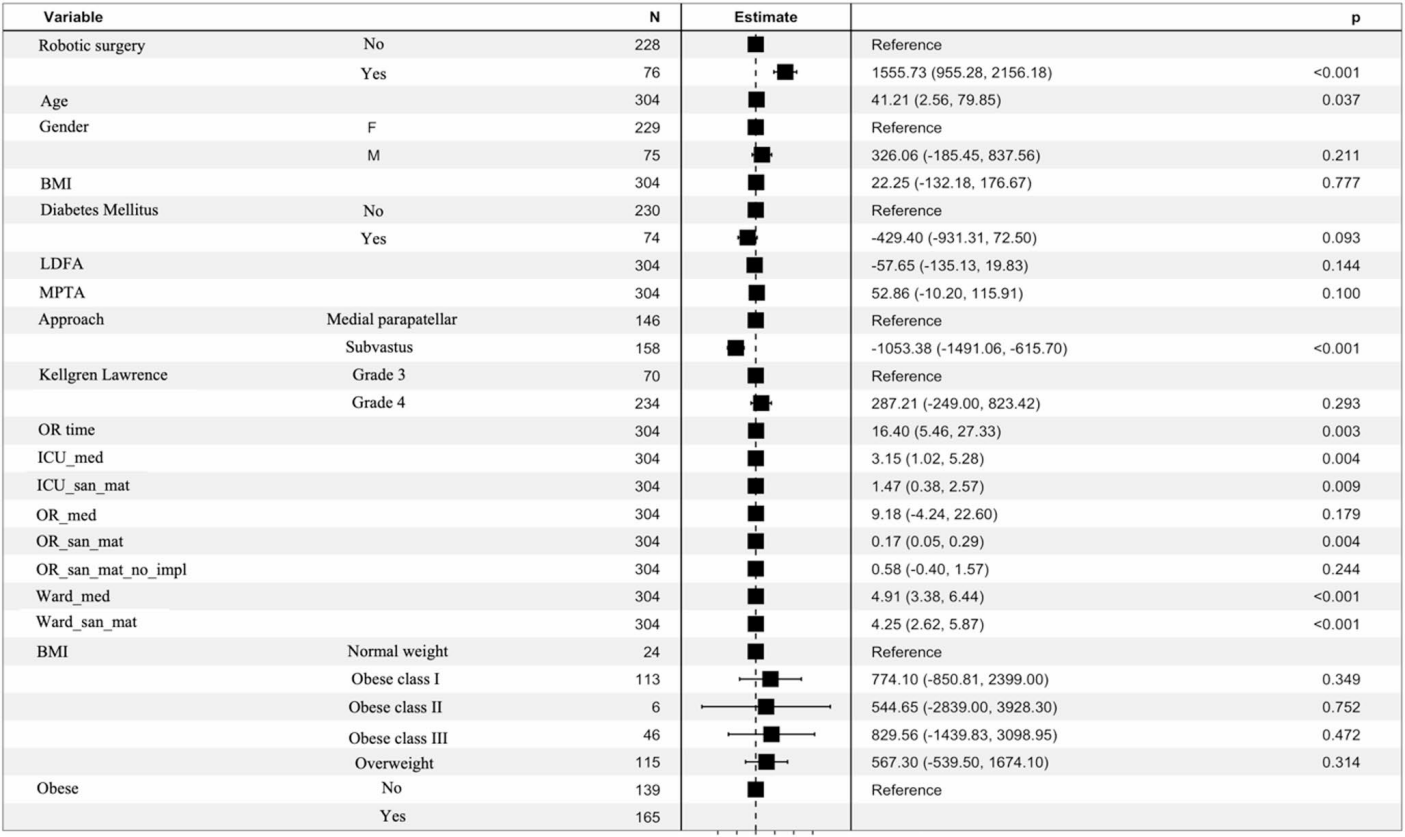
Forest plot of main regression coefficients

The estimated effects are summarized in Fig. 3, along with their corresponding confidence intervals. Based on OLS estimates, higher costs were significantly associated with ward medication (β = 4.91, *P* < 0.01), robotic intervention (β = 1555.73, *P* < 0.01), and ward sanitary materials (β = 4.25, *P* < 0.01). In contrast, the subvastus approach was associated with lower costs (β = −1053.38, *P* < 0.01). Additional significant predictors included, among others, operating room time (β = 16.40, *P* = 0.002), ICU medication (β = 3.15, *P* = 0.003), and ICU sanitary materials (β = 1.47, *P* = 0.009). Model performance shows moderate explanatory power (R² ≈ 43%).

### Cost breakdown across hospitalization components

To further examine how costs are distributed across different stages of care, separate multiple linear regression models were estimated for each cost component (Table 2 in the Appendix). These models began with the complete set of predictors available and then the final relevant variables were selected using stepwise regression. Where appropriate, log-transformed dependent variables were used to account for non-normal distributions.

Across cost categories, the explanatory power of the models was generally modest (adjusted R² < 10%), indicating that individual cost components are influenced by a wider range of unobserved factors. Robotic intervention showed heterogeneous associations across cost components. It was positively associated with operating room medication costs (log-transformed; β = 0.112, *p* = 0.012) and negatively, although not statistically significant, with operating room sanitary material costs (β = −2942.32, *p* = 0.052).

Patient-level characteristics also showed differential effects. Body mass index was negatively associated with ICU sanitary material costs (β = −0.03, *p* = 0.003), while diabetes mellitus was associated with reduced ICU sanitary material costs (β = −0.14, *p* = 0.04) but increased ward medication costs (β = 41.41, *p* = 0.035). Age influenced specific cost categories, such as bloc materials excluding prosthesis (β = 0.005, *p* = 0.05), while longer hospital duration was associated with increased ward medication costs (β = 0.97, *p* = 0.027). Notably, while the model for total hospitalization costs captured a moderate proportion of variance, models for individual cost components showed substantially lower explanatory power, suggesting that total cost is more predictable than its underlying components.

## Discussion

This study provides a structured view of hospitalization costs following total knee arthroplasty by comparing rTKA with mTKA and by distinguishing between overall cost determinants and the underlying cost components. The study uses a large patient dataset and a combination of LASSO regression and multiple linear OLS models. Our findings confirm that hospitalization costs are influenced by a complex interplay of clinical, procedural, and patient-related factors, some of which align with expectations, while others challenge conventional assumptions.

The most consistent finding was that rTKA increased total hospitalization costs, largely through higher expenditures for medication, OR time, and certain material categories. This is in agreement with prior reports indicating that robotic systems add both direct costs (e.g., consumables, disposables) and indirect costs through prolonged operating time in the learning phase [15, 16]. According to Rajan et al., the higher per-case cost of rTKA can be amortized through reduced annual revision rates and better postoperative QoL, particularly when centers perform more than 24 cases annually [16. While higher initially, costs may be neutralized over time by decreased complications or revisions, a premise that requires longitudinal confirmation on long term. Our results are consistent with these findings in terms of complications or readmissions. Given the very low number of events, these findings should be interpreted descriptively, as the study was not powered to detect significant differences in complication rates. In the rTKA group, only one incident was reported during the 90-day follow-up without injury and unrelated to the stability of the operated knee while in the mTKA group three complications were recorded. One case of wound dehiscence necessitated hospital readmission, therefore adding additional costs.

Unexpectedly, contrary results arise in the hospitalization days in the rTKA group. Generally considered a technique that facilitates quicker recovery, it was associated with an increase in hospitalization days. These findings are contradictory to the ones reported in literature where patients that underwent rTKA, experienced faster recovery with shorter LOS [17–19]. For example, Archer et al. reported discharge within ≤ 1 day after rTKA versus a mean LOS of ~ 2.3 days for the mTKA group [17]. In our cohort, these findings likely reflect center-specific implementation factors rather than an intrinsic disadvantage of the robotic technique. During the study period, the robotic program was in an early adoption phase, with additional time required for system setup, anatomical registration, tracker placement, and workflow familiarization, which may explain the longer operative times. Furthermore, postoperative discharge pathways were not specifically optimized for robotic cases, and identical discharge criteria were applied to both groups. Therefore, length of stay was likely influenced more by institutional logistics, rehabilitation availability, and perioperative organization than by the surgical platform itself. This highlights the importance of contextual factors in interpreting cost outcomes.

Other notable findings highlight the significant influence of patient-related variables such as age, BMI, and diabetes on specific cost components. Higher BMI and advanced age were associated with increased use of materials and medications, consistent with previous literature showing that these factors drive higher perioperative resource consumption. Maxwell et al. reported that elevated BMI was linked to increased index and 90-day episodic costs in patients undergoing primary TKA, while George et al. found that higher BMI was also associated with greater complication and readmission rates after TKA [20, 21]. In our study, the single readmission case occurred in the mTKA group and involved a patient with a BMI of 39 kg/m², reinforcing these observations. Diabetes, on the other hand, appeared to reduce some ICU-related costs but increase ward medication expenses, suggesting variations in postoperative care patterns. These findings align with prior research indicating that diabetes is associated with higher overall hospitalization costs [22, 23].

The relatively modest explanatory power of our models for some cost subcategories (adjusted R² < 10%) underscores the variability in hospital billing structures and the influence of unmeasured factors such as staff experience, intraoperative complications, or rehabilitation protocols. In contrast, the total cost model achieved an adjusted R² of ~ 43%, which, while moderate, is comparable to other healthcare cost analyses where multiple uncontrollable factors are at play.

Key limitations include the retrospective, single-center design and a short 90-day follow-up, which may miss later complications, revisions, and costs. In addition, the study does not represent a full economic evaluation, as capital investment, maintenance costs of robotic systems, and broader system-level costs were not included. Also, the article is limited by categorical cost reporting and the absence of detailed itemized reports, constrained by the dataset’s comprehensiveness. Future studies should focus on long-term cost-analysis, incorporating quality-adjusted life years (QALYs), revision rates, and functional outcomes to provide a more comprehensive assessment of the economic value of robotic-assisted TKA. Another limitation of the present study is that surgeon-specific proficiency and learning curve effects could not be fully quantified, particularly for robotic-assisted TKA. Although all procedures were performed by experienced high-volume arthroplasty surgeons, robotic case exposure varied among the participating surgical teams, with a relatively limited and uneven distribution of robotic procedures per team. Consequently, a reliable subgroup analysis according to individual surgeon experience or progression along the robotic learning curve was not feasible.

A final limitation of this study is that robotic-assisted TKA was performed using a kinematic alignment philosophy, whereas manual TKA followed conventional mechanical alignment principles, reflecting routine institutional practice. Although this may represent a potential confounder, the study focused primarily on perioperative costs and 90-day complications, outcomes less likely to be substantially influenced by alignment strategy compared with long-term implant survivorship or late mechanical complications.

Beyond direct financial costs, sustainability has emerged as an additional consideration in arthroplasty care. As highlighted by Regmi et al., orthopaedic surgery is associated with substantial waste generation, resource consumption, and carbon emissions, supporting the need to evaluate new technologies not only in economic terms but also from an environmental perspective [24]. In this context, robotic platforms may increase resource utilization through capital equipment, maintenance, energy consumption, and disposable components, while potential gains in precision and efficiency may offset part of this burden. Future comparative studies should therefore integrate both economic and environmental outcomes through dedicated lifecycle assessments.

## Conclusions

This analysis shows that cost differences between robotic and conventional TKA are not fully attributable to the surgical approach. Costs reflect an interplay of surgical technique, patient characteristics, and institutional practices, producing a varied cost profile. Although robotic TKA is currently associated with higher hospitalization costs, careful optimization of perioperative protocols, including medication management, operating room efficiency, and resource allocation may reduce these expenses without compromising patient outcomes.

## Appendix

See appendix Table 2.

**Table 2:** Linear models to predict cost components

Model	Log(ICU medication)	Log(ICU sanitary materials)	Log(OR medication)	OR sanitary materials	Log(OR materials no impl)	Log(Ward sanitary materials)	Ward medication
Intercept	4.83*** (*p* < 0.001)	5.03*** (*p* < 0.001)	3.08*** (*p* < 0.001)	6189.44*** (*p* < 0.001)	5.91*** (*p* < 0.001)	4.83*** (*p* < 0.001)	260.91*** (*p* < 0.001)
Robotic (yes)	-	-0.69 (*p* = 0.151)	0.112* (*p* = 0.012)	-2942.32 (*p* = 0.052)	-	-	-43.73* (*p* = 0.048)
Age	-	0.01 (*p* = 0.08)	-	-	0.005* (*p* = 0.05)	-	-
Sex	-	-	0.077 (*p* = 0.087)	-	-	-	-
BMI	-	-0.03** (*p* = 0.003)	-	16.44 (*p* = 0.53)	-	-0.026 (*p* = 0.128)	-
Obese No Yes	-	-	-	-	-	Reference 0.256 (*p* = 0.08)	-
Diabetes Mellitus (yes)	-0.13 (*p* = 0.09)	-0.14* (*p* = 0.04)	-	-	-	-	41.41* (*p* = 0.035)
Duration	-	0.002 (*p* = 0.13)	-	-	-	-	0.97* (*p* = 0.027)
Approach Standard Subvast	-	-	Reference -0.07 (*p* = 0.076)	Reference 307.31 (*p* = 0.101)	Reference 0.05 (*p* = 0.11)	Reference -0.16 (*p* = 0.08)	-
KL index	-	-	-	-390.71 (*p* = 0.101)	-	-	-
BMI*Robotic	-	0.022 (*p* = 0.141)	-	119.55* (*p* = 0.014)	-	-	-
R^2^/Adj R^2^	0.7%/0.5%	5.7%/4.1%	3.2%/2.4%	8.3%/7.04%	1.8%/ 1.3%	1.5%/0.7%	2.6%/1.8%

## Data Availability

The datasets used and/or analyzed during the current study are stored in the institutional database and were extracted from the hospital management system. Due to institutional and privacy restrictions, these data are not publicly available but are available from the corresponding author on reasonable request.
